# Increased central common drive to ankle plantar flexor and dorsiflexor muscles during visually guided gait

**DOI:** 10.14814/phy2.13598

**Published:** 2018-02-06

**Authors:** Peter Jensen, Nicole Jacqueline Jensen, Cecilie Ulbæk Terkildsen, Julia T. Choi, Jens Bo Nielsen, Svend Sparre Geertsen

**Affiliations:** ^1^ Department of Nutrition, Exercise and Sports University of Copenhagen Copenhagen Denmark; ^2^ Department of Neuroscience University of Copenhagen Copenhagen Denmark; ^3^ Department of Kinesiology University of Massachusetts Amherst Massachusetts; ^4^ Elsass Institute Charlottenlund Denmark

**Keywords:** coherence, EMG, locomotion, visually guided walking

## Abstract

When we walk in a challenging environment, we use visual information to modify our gait and place our feet carefully on the ground. Here, we explored how central common drive to ankle muscles changes in relation to visually guided foot placement. Sixteen healthy adults aged 23 ± 5 years participated in the study. Electromyography (EMG) from the Soleus (Sol), medial Gastrocnemius (MG), and the distal and proximal ends of the Tibialis anterior (TA) muscles and electroencephalography (EEG) from Cz were recorded while subjects walked on a motorized treadmill. A visually guided walking task, where subjects received visual feedback of their foot placement on a screen in real‐time and were required to place their feet within narrow preset target areas, was compared to normal walking. There was a significant increase in the central common drive estimated by TA‐TA and Sol‐MG EMG‐EMG coherence in beta and gamma frequencies during the visually guided walking compared to normal walking. EEG‐TA EMG coherence also increased, but the group average did not reach statistical significance. The results indicate that the corticospinal tract is involved in modifying gait when visually guided placement of the foot is required. These findings are important for our basic understanding of the central control of human bipedal gait and for the design of rehabilitation interventions for gait function following central motor lesions.

## Introduction

When we walk in a challenging environment, we rely on visual information to navigate and circumvent obstacles in the environment (Drew 1991; Patla and Vickers 1997, 2003; Gerin‐Lajoie et al. 2005, 2008; McFadyen et al. 2007; Marigold 2008; Drew and Marigold 2015; Maeda et al. 2017). Visually guided gait adjustments are made in anticipation of an encounter with an obstacle in our path (Marigold 2008; Matthis and Fajen 2014). For example, we may alter step length to ensure that our supporting foot is optimally placed to help the other foot over the obstacle (Mohagheghi et al. 2004; Drew et al. 2008; Drew and Marigold 2015). Ongoing visual information from the environment also influences the rhythmicity of our gait as shown by a significant effect of changes in optic flow on step frequency in subjects walking on a self‐driven treadmill (Prokop et al. 1997; Lamontagne et al. 2007).

Experiments in the cat have demonstrated that the motor cortex plays an important role in visually guided gait modifications (Drew and Marigold 2015). Corticospinal neurons directly contribute to the control of paw placement during walking on a horizontal ladder where the cat has to place its paw carefully on the rungs of the ladder (Drew et al. 1996). Corticospinal neurons in the cat are also involved in controlling the lift of the paw over an obstacle during visually guided treadmill walking (Drew et al. 1996), by modifying the phase and magnitude of synergistic muscles (Krouchev and Drew 2013). Posterior parietal areas play a key role in integrating the visual and sensory information, which is used by the motor cortex to control appropriate paw placement and paw lift (Drew et al. 2008; Drew and Marigold 2015).

In humans, experimental demonstration of how the motor cortex contributes to visually guided locomotion is limited to one study that used transcranial magnetic stimulation (TMS) to probe corticospinal excitability during precision walking. Schubert et al. (1999) demonstrated that TMS‐evoked responses in the TA muscle were facilitated when subjects were asked to place their feet on visual targets on a treadmill. This may indicate that the human motor cortex plays an important role in the control of foot placement as in the cat.

Synchronization of electromyography (EMG) activity recordings during voluntary muscle contractions is thought to reflect corticospinal input to a given set of muscles (Farmer et al. 1993). In walking, intramuscular coherence between a pair of EMG signals from the same leg muscle (e.g., proximal and distal TA) has been found in the beta and gamma frequency bands (Halliday et al. 2003; Petersen et al. 2010). Intermuscular coherence between different leg muscles also contains both beta and gamma frequency bands during walking (Halliday et al. 2003; Norton and Gorassini 2006). Evidence to support that EMG‐EMG coherence are at least in part modulated by the motor cortex via the corticospinal tract can be derived from studies of injuries in the central nervous system where coherence in the beta and gamma frequency bands are absent or markedly reduced in patients with stroke and spinal cord injury (Hansen et al. 2005; Nielsen et al. 2008; Barthelemy et al. 2010). In addition, EMG‐EMG coherence for both the upper and lower extremities increase with maturation of the corticospinal tract (Farmer et al. 2007; Petersen et al. 2010). Furthermore, during walking the corticomuscular coherence between electroencephalography (EEG) signals and the TA EMG has been shown to be localized roughly over the leg motor cortex, with maximal coupling at or near the electrode Cz (Petersen et al. 2012). This corticomuscular EEG‐EMG coherence is significant in the 24–40 Hz frequency band during walking (Petersen et al. 2012). It is not yet clear how EMG‐EMG and EEG‐EMG coherence would change when subjects make visually guided adjustments during walking. An increase in coherence will be indicative of additional common cortical input needed to guide precise foot placement.

The presence of EMG‐EMG and EEG‐EMG coherence during gait allows us to examine the functional role of the motor cortex and corticospinal tract, by comparing coherence during walking with and without visual feedback. We aimed in this study to investigate changes in intra‐, intermuscular, and corticomuscular coherence in ankle muscles related to visually guided foot placement during treadmill locomotion. Intra‐ and intermuscular coherence in the beta and gamma bands during treadmill locomotion have been shown in all likelihood to reflect corticospinal input to the muscles (Halliday et al. 2003; Hansen et al. 2005; Nielsen et al. 2005; Barthelemy et al. 2010). Thus, by calculating the EMG‐EMG and EEG‐EMG coherence it is possible to investigate the central common drive to the motoneurones without perturbing the system and with no discomfort for the subjects. We hypothesized that coherence would be larger when subjects were asked to visually control the placement of their feet on the treadmill as compared to normal treadmill walking without requirement of accurate foot placement.

## Methods

### Ethical approval

Sixteen healthy adult subjects (10 female/6 male, range 18–35, mean 23 ± 5 years) gave their written, informed consent to participate in the study. The study was approved by the ethics committee for the Capital Region of Denmark (Approval No. H‐16021214) and was performed in accordance with the Declaration of Helsinki.

### Motion capture

A total of 14 reflective markers (size 12 mm diameter) were placed bilaterally on the lateral side of the fifth metatarsal, the heel, the lateral malleolus, the lateral articular line of the knee, the greater trochanter, the anterior superior iliac spine, and the acromion. 3‐D marker data were collected with a six‐camera ProReflex motion capture system (Qualisys, Gothenburg, Sweden) at 100 Hz. The markers were identified using the AIM module in the Qualisys Track Manager (QTM) software.

### Experimental setup & visual feedback

Subjects performed two 5‐min walking tasks at the same predetermined treadmill speed: 1) normal walking and 2) visually guided walking.

During normal walking, subjects walked with open eyes and were asked to look straight ahead at an X on the wall in front of them, but received no information of foot placement. During visually guided walking, subjects received visual feedback of their foot placement on a screen in real‐time and were required to adjust their step length to hit virtual visual targets. Stepping targets and foot position were projected on a screen in front of the treadmill. The projector (TDP‐T 355; Toshiba, Tokyo, Japan) was mounted on the ceiling 148 cm from the screen, which gave a screen size of 125 cm by 167 cm. The target locations were manipulated step by step with real‐time visual feedback of each step. A custom‐made computer program controlled the position of the targets, while the QTM real‐time server provided the position of the foot (i.e., fifth metatarsal marker) during the walking task.

**Figure 1:**
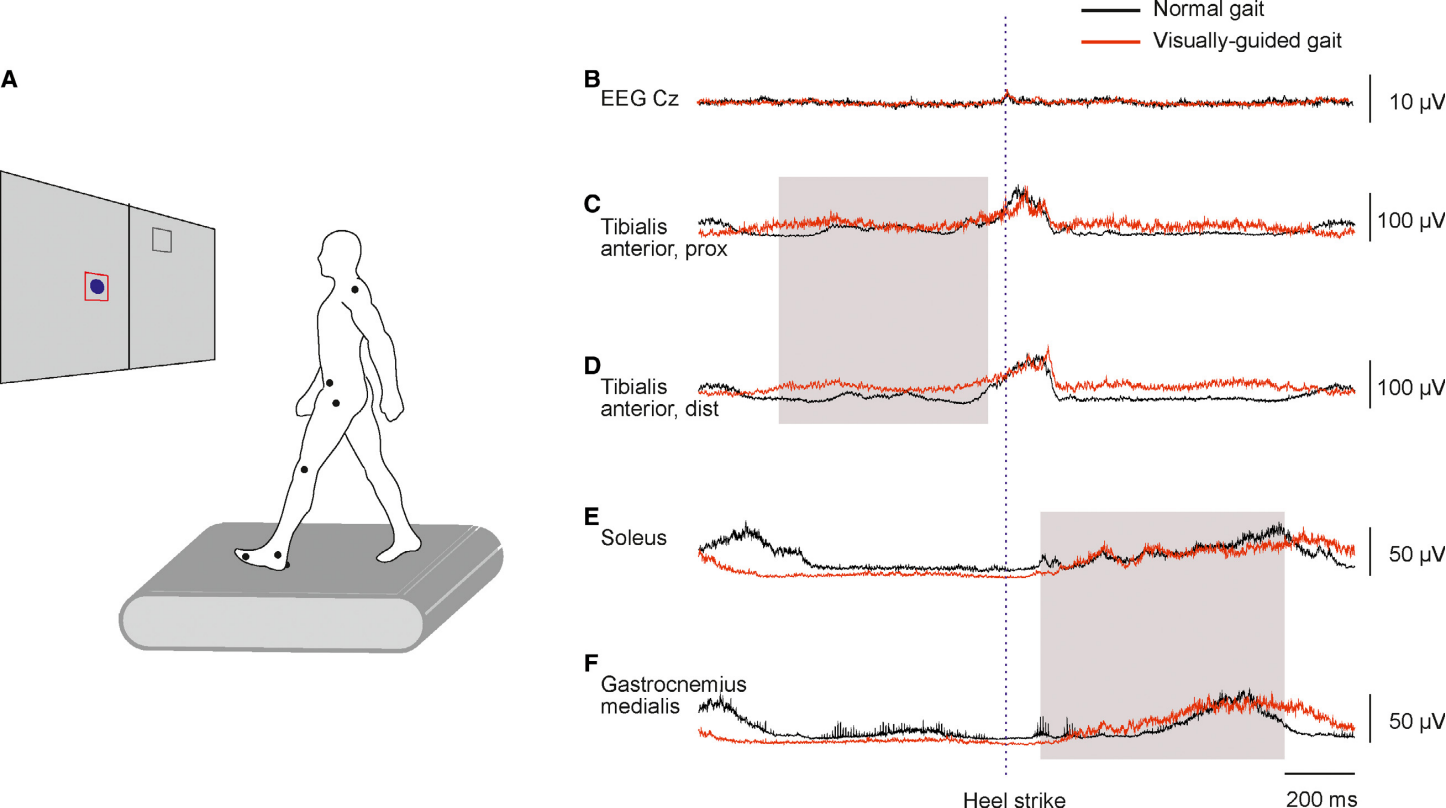
The visually guided walking task. (A) Subjects were instructed to adjust their step length to hit virtual visual targets while walking on a motorized treadmill. Stepping targets and foot position were projected on a screen in front of the treadmill. The position of the foot of the swing leg was displayed as an 8 cm diameter blue circle and the targets were shown as 16 cm x 16 cm open red squares on the screen. (B–F) EEG and EMG traces from one representable subject during normal (black) and visually guided gait (red). The vertical dashed line represents the time of heel strike. The gray‐shaded areas show the time‐periods used for coherence analyses. EMG, electromyography; EEG, electroencephalography.

The position of the foot of the swing leg was displayed as an 8 cm diameter blue circle. And the targets were shown for each step as 16 × 16 cm open red squares on the screen (Fig. 1A). The required step length was visually represented on every step by the vertical distance between two targets on the left and right side, respectively. Subjects were instructed to step on the targets as accurately as possible. A successful hit was one where the center of the foot (circle) lay within 6 cm of the center of the target (square) after heel‐strike. The score (# hits) was calculated and displayed live on the screen. The final hit rate (in %) was calculated as the number of successful hits divided by the total number of steps in the 5 min session.

The step length and treadmill speed were normalized to each subject's leg length. The step length was defined as 2/3 of the leg length measured from the left greater trochanter to the left lateral malleolus. The treadmill speed was then determined by multiplying the subject's step length with 3.9 in order to match the cadence of 80 steps per minute between subjects (see Choi et al. (2016)). Prior to the visually guided walking, subjects were carefully introduced to the task. They were asked to walk normally and to only adjust their step length in order to hit the virtual targets. All experiments were closely supervised by the test leaders.

### Data collection

After careful preparation of the skin (shaving and abrasion), pairs of surface EMG electrodes (Ambu Blue sensor NF‐00‐A/12, Ambu A/S Ballerup. Recording area 0.5 cm^2^, interelectrode distance 2 cm) were placed on the left distal and proximal ends of the TA, Soleus (Sol), and medial Gastrocnemius (MG) muscles. EEG activity was recorded through a pair of bipolar silver electrodes placed at the vertex (Cz) and 5 cm frontal to Cz. The ground electrode was placed on the left elbow.

EMG (amplified x1000, Zerowire, Aurion, Italy) and EEG signals (amplified x10000, custom‐built amplifiers) were sampled at 2000 Hz (Micro 1401 and Spike 2, Cambridge Electronic Design, UK) and stored on a computer for later offline analysis. A footswitch sensor (Zerowire) was placed under the left heel to record the time of heel strike (Fig. 1B–F).

### Coherence analyses

The time and frequency domain analysis was performed on EEG and surface EMG signals to investigate corticomuscular (Cz‐TA and Cz‐Sol), intramuscular (TA‐TA), and intermuscular coherence (Sol‐MG). Neurospec 2.0 software was used for all coherence analysis.

The EMG signals were rectified to maximize the information on the timing of the action potentials of individual motor units while suppressing information on the shape of the action potential waveform (Halliday and Farmer 2010). EEG signals and rectified EMG signals were normalized to have unit variance (Halliday and Rosenberg 2000). Normalized EEG and rectified and normalized EMG signals are assumed to be realizations of stationary zero mean time series denoted by x and y. To estimate the average autospectra ƒ_xx_(*λ*) and ƒ_yy_(*λ*) and cross spectra ƒ_xy_(*λ*) discrete Fourier transform was applied to the signals. Frequency domain analysis of correlation between EEG‐EMG and EMG–EMG was then made. Coherence estimates are bounded measures of association defined over the range of [0, 1]; cumulant density estimates are not bounded, and phase is defined over the range [−*π*, +*π*]. For the present data, coherence estimates quantify the strength and range of frequencies of common rhythmic synaptic inputs distributed across the motoneuron pool. The cumulant density function provides an unbounded time‐domain representation of the correlation structure analogous to the cross correlogram.

The step cycles were identified using the footswitch sensor and the windows for analysis of coherence were based on visual inspection of the EMG activity in relation to heel strike. A 700‐ms window from 100 to 800 ms after the heel strike was used for the stance phase/push off (Cz‐Sol and Sol‐MG coherence), and a 600‐ms window from −650 to −50 ms before heel strike was used for the swing phase/foot clearance (Cz‐TA and TA‐TA coherence). Thus, the coherence analysis was based on averaging data segments with the same relative timing to the footswitch. Time frequency estimates were made using a sliding data segment of 500 ms with a variable offset from each heel strike with increments of 10 ms, which provided a resolution of 4 Hz in the spectral estimates. Heat maps were constructed for the interaction between EEG‐EMG and EMG–EMG. In the heat maps, the *x*‐axis refer to the time from the offset to the start of each data segment in respect to heel strike.

## Statistics

Based on the assumption of statistical independence significance is set by an inclusion of an upper 95% confidence limit for the coherence plots and an upper and lower 95% confidence limits in the cumulant density plots (Halliday et al. 1995). This provides a measurement of the common input to the two pools of motoneurons and thus is a noninvasive way of analyzing changes in the direct drive from the motor cortex to the working muscles.

The final analysis was made on the pooled data from the EEG and the surface EMG from the two muscles (Halliday and Rosenberg 2000). Changes in the correlation structure between two different tasks can be ascertained by undertaking a chi^2^ extended difference of coherence test on the tasks to be compared. The resulting chi^2^ difference test thus provides a metric of amount of pooled coherence difference at each frequency between the two tasks (Farmer et al. 2007). The difference in coherence between normal and visually guided walking was evaluated using a chi^2^ extended difference test (Amjad et al. 1997).

The amount of coherence was in addition quantified by calculating the logarithmic value of the cumulative sum (i.e., area) within three frequency bands: alpha: 5–15 Hz, beta: 15–35 Hz and gamma: 35–60 Hz. A paired t‐test was then used to investigate possible differences between normal and visually guided walking in these frequency bands. To investigate possible correlations between hit rate during visually guided walking and the amount of coherence in the alpha, beta and gamma frequency bands, respectively, we used Pearson product moment correlations. Statistical significance was given for *P*‐values smaller than 0.05. Data are presented as means ± standard deviation (SD) unless reported otherwise.

## Results

In both walking tasks, subjects walked at a treadmill speed of 2.2 ± 0.1 km/h with a step length of 0.56 ± 0.04 m. The step frequency was similar in normal (80 ± 11 steps/min) and visually guided walking (79 ± 6 steps/min) and not significantly different (*P *>* *0.3).

During visually guided walking, subjects hit the target 327 ± 33 out of 410 ± 17 steps corresponding to a hit rate of 82 ± 6%.

### Time‐frequency analysis of coherence over the gait cycle

**Figure 2:**
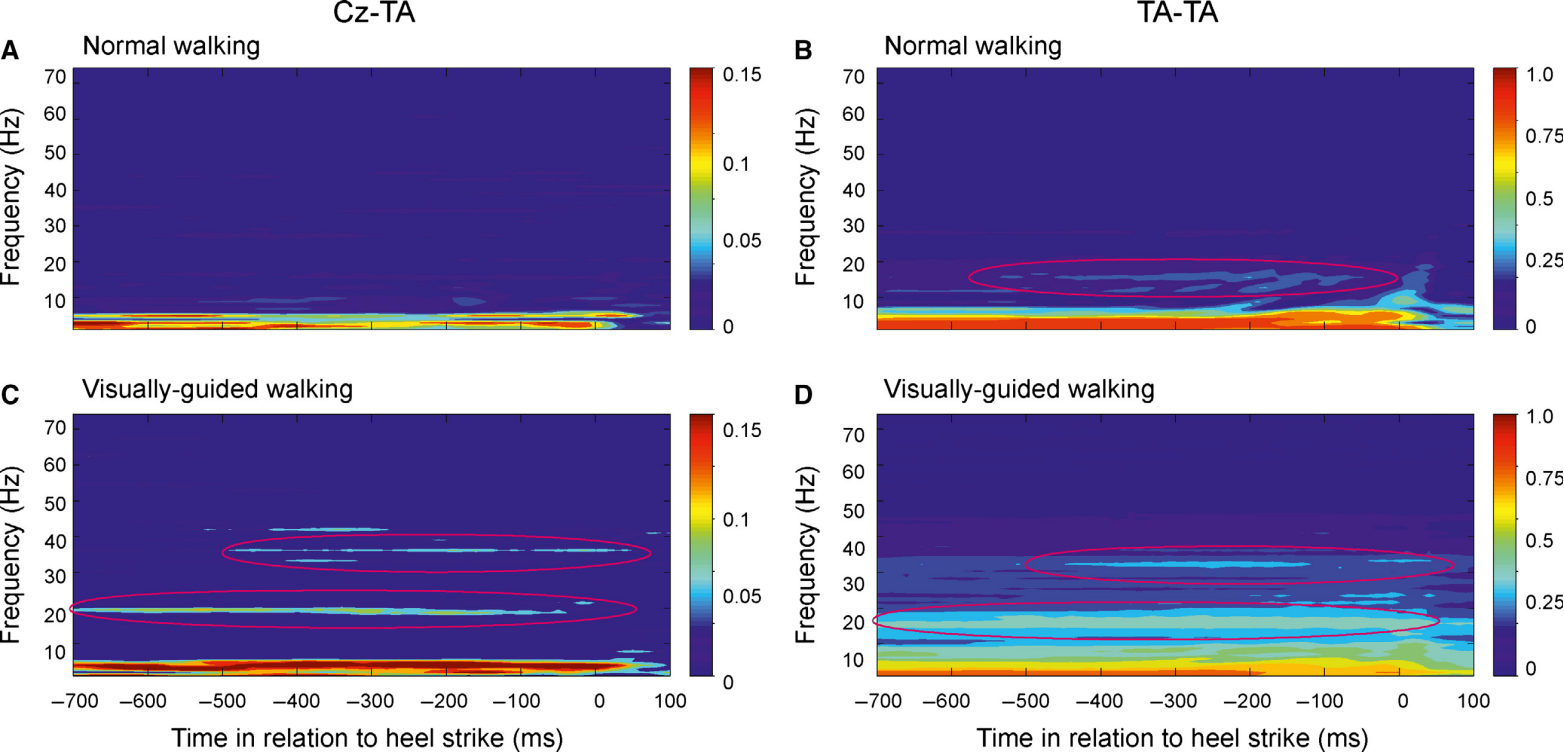
Time‐frequency plot of corticomuscular (Cz‐TA) and intramuscular coherence (TA‐TA) from a single subject during normal and visually guided walking. (A–B) During normal walking, significant estimates of TA‐TA coherence was observed in the beta frequency band (15–35 Hz; see red circle in B). (C–D) During visually guided walking, significant estimates of both Cz‐TA and TA‐TA coherence was observed in the beta (15–35 Hz) and gamma frequency bands (35–60 Hz) for offset values between −700 and −50 ms (see red circles in C and D). The very high and significant coherence observed at frequencies below 10 Hz is assumed to be produced, in part, by the common envelope of the EMG activity during swing (Halliday et al. 2003). EMG, electromyography; TA, Tibialis anterior.

Figure 2 shows the time‐frequency coherence analysis between TA‐TA and Cz‐TA from a single subject. For TA‐TA, some beta coherence at 15–20 Hz was seen during normal walking from around −550 to −50 ms prior to heel strike (Fig. 2B), but during visually guided walking this was much clearer and low gamma coherence could also be observed (Fig. 2D). For Cz‐TA, only low‐frequency alpha coherence was evident during normal walking (Fig. 2A), but clear beta and low gamma coherence could also be observed during visually guided walking (Fig. 2C).

### Pooled coherence estimates

**Figure 3:**
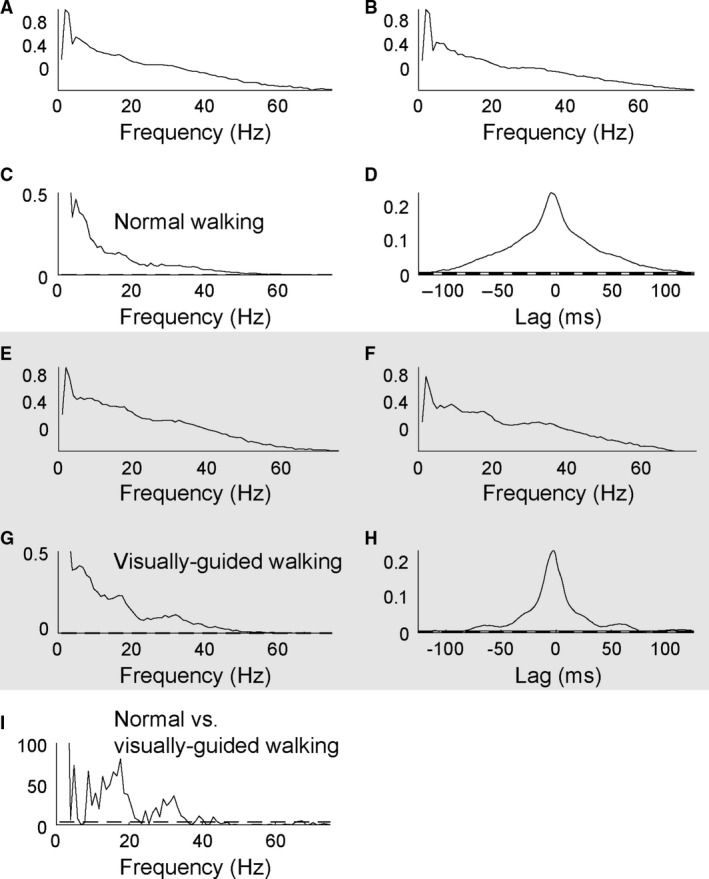
Pooled intramuscular TA‐TA coherence during normal and visually guided walking. Autospectra (0–75 Hz) of the proximal and distal TA electrodes during normal (A and B) and visually guided walking (E and F). Coherence at frequencies between 1 and 75 Hz calculated between TA rectified EMGs during normal (C) and visually guided walking (G). Cumulant densities (range ± 125 ms) associated with the coherence during normal (D) and visually guided walking (H). *χ* ^2^ analysis of the difference in TA‐TA coherence in the two walking tasks (I). The dashed horizontal lines denote the upper 95% confidence limit based on the assumption of independence. Note that there is significantly more TA‐TA coherence in both the beta and gamma range during visually guided walking. EMG, electromyography; TA, Tibialis anterior.

Figure 3 shows the pooled TA‐TA coherence during normal (Fig. 3A–D) and visually guided walking (Fig. 3E–H). While subjects had significant TA‐TA coherence during normal walking (Fig. 3C), there was significantly more TA‐TA coherence in both the beta and gamma frequency bands during visually guided walking (Fig. 3G and I). Cumulant densities constructed from the rectified EMG data are shown in Fig. 3D and H for normal and visually guided walking. Note that the central peak around 0 ms, indicating synchronization between the two rectified EMGs, is narrower during the visually guided walking.

A similar pattern with significantly larger peaks around 15–20 Hz and around 35–40 Hz during visually guided walking was found for the Sol‐MG coherence. The central peak around 0 ms was also narrower during the visually guided walking indicating a more synchronized input to MG and Sol motor units during this task (data not shown).

### Quantification of EMG‐EMG and EEG‐EMG coherence

**Figure 4:**
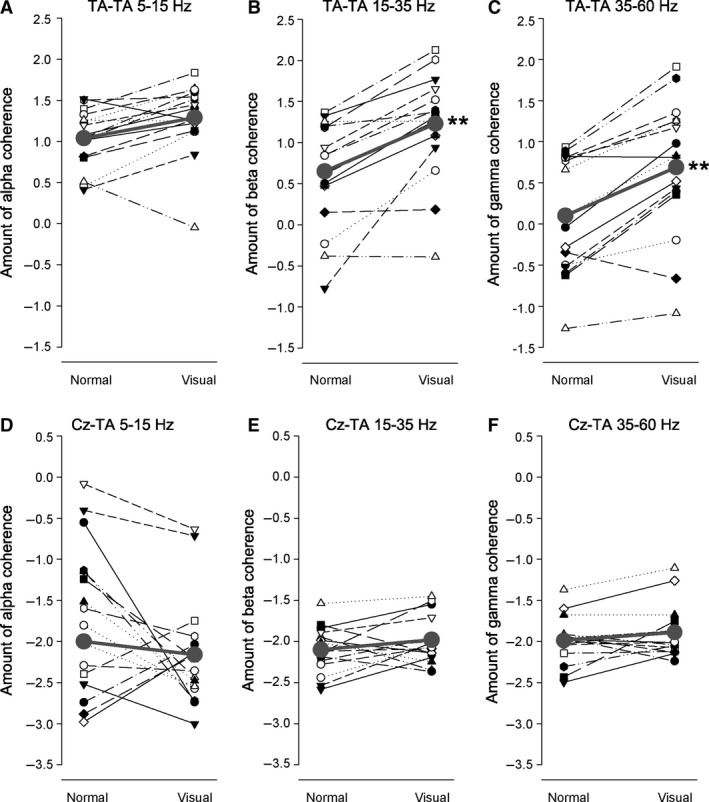
Intramuscular (TA‐TA) and corticomuscular (Cz‐TA) coherence during normal and visually guided walking. A‐C shows the amount of alpha (A), beta (B), and gamma (C) intramuscular coherence between TA‐TA during normal and visually guided gait for each subject. D–F shows the amount of alpha (D), beta (E), and gamma (F) corticomuscular coherence between Cz‐TA during normal and visually guided gait. The thick gray line shows the average amount of coherence in each task. **Significant difference (*P *<* *0.01) in the amount of coherence during normal and visually guided gait. TA, Tibialis anterior.

**Figure 5:**
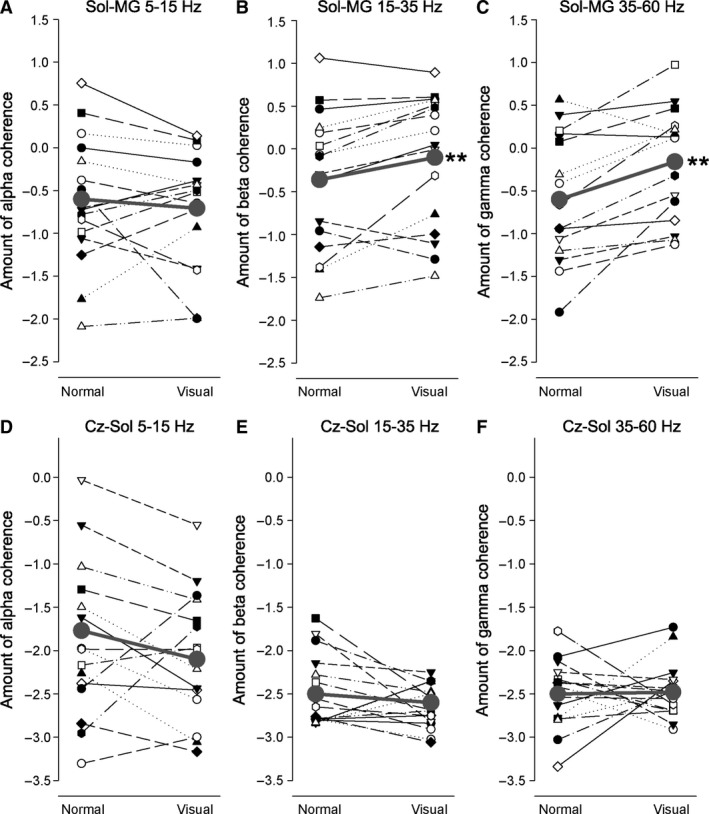
Intermuscular (Sol‐MG) and corticomuscular (Cz‐Sol) coherence during normal and visually guided walking. (A–C) Shows the amount of alpha (A), beta (B), and gamma (C) intermuscular coherence between Sol‐MG during normal and visually guided gait for each subject. (D–F) Shows the amount of alpha (D), beta (E), and gamma (F) corticomuscular coherence between Cz‐Sol during normal and visually guided gait. The thick gray line shows the average amount of coherence in each task. **Significant difference (*P *<* *0.01) in the amount of coherence during normal and visually guided gait. MG, medial Gastrocnemius; Sol, Soleus.

Figures 4 and 5 shows the amount of EMG‐EMG and EEG‐EMG coherence during normal and visually guided walking for each subject. There was a statistically significant increase in both TA‐TA (Fig. 4B–C) and Sol‐MG (Fig. 5B–C) coherence in the beta and gamma frequency bands (all *P *<* *0.01), but no difference in the alpha band (*P *>* *0.1; Fig. 4A and 5A). There was no statistically significant change in EEG‐EMG coherence in any frequency band for any of the muscles (*P *>* *0.2; Fig. 4D–F and Fig. 5D–F).

**Figure 6:**
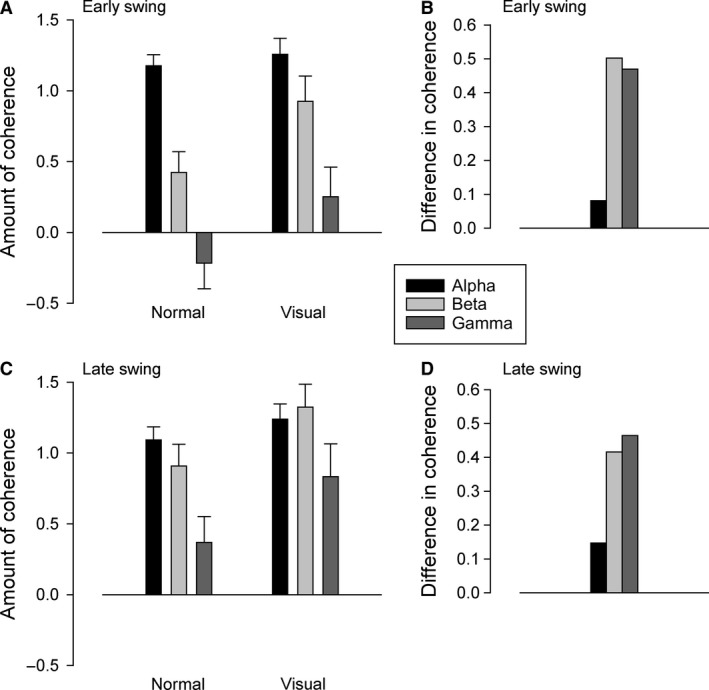
Coherence in early and late swing. Amount of TA‐TA alpha (black), beta (light gray), and gamma coherence (dark gray) in the early (−650 to −350 ms) and late part of the swing phase (−350 to −50 ms) during normal and visually guided walking. Note that beta and gamma coherence was increased during visually guided walking in both early and late swing. TA, Tibialis anterior.

TA EMG often shows two clear peaks during a gait cycle; in the early and late part of the swing phase. To investigate if the increased beta and gamma coherence observed during visually guided walking was linked specifically to one of these peaks, we analyzed the amount of TA‐TA coherence in the early (−650 to −350 ms) and late (−350 to −50 ms) part of the swing phase separately for the two walking tasks. However, as shown in Figure 6, TA‐TA coherence was increased in both early (*P *<* *0.01) and late (*P *<* *0.01) swing to a similar extent.

There was no significant correlation between the hit rate of the individual subjects during visual‐guided walking and the amount of either intramuscular, intermuscular, or corticomuscular coherence in any of the three frequency bands. However, the correlation coefficient for the relation between corticomuscular coherence in the beta band and the hit rate approached significance (*r *=* *0.45, *P *=* *0.09).

## Discussion

We have demonstrated in this study that intramuscular, intermuscular, and corticomuscular coherence measured from ankle dorsiflexors and plantar flexors increase during treadmill walking when subjects use visual information to step on targets. This is in line with animal studies showing increased corticospinal activity during visually guided walking and suggests that the motor cortex is involved in steering foot placement with the help of visual information during gait.

Intra‐ and intermuscular coherence was pronounced in both the alpha, beta, and gamma frequency bands for both dorsiflexors in the swing phase and plantar flexors in the stance phase during ordinary treadmill walking similar to what has been reported previously (Halliday et al. 2003; Norton and Gorassini 2006). Coherence increased significantly in the beta and gamma bands in relation to visually guided walking without any changes in the alpha band. Previous findings have suggested that intra‐ and intermuscular coherence in the beta and gamma bands during walking is related to corticospinal activity, since coherence at similar frequencies are 1) observed for paired EEG and EMG recordings (Petersen et al. 2012; Storzer et al. 2016; Winslow et al. 2016), 2) greatly reduced following lesion of the corticospinal tract (Hansen et al. 2005; Nielsen et al. 2008; Barthelemy et al. 2010; Petersen et al. 2013) and 3) increased in relation to maturation of the corticospinal tract (Farmer et al. 2007; James et al. 2008; Petersen et al. 2010). In line with this, corticomuscular coherence in the beta and gamma frequency bands also increased during visually guided walking although it did not reach statistical significance. One reason for this may be that we failed to determine any significant corticomuscular coherence in the majority of subjects. In those subjects in whom significant corticomuscular coherence was observed during normal treadmill walking, we did observe clear and significant increases in coherence during visually guided walking. Our failure to determine significant corticomuscular coherence during normal walking may be related to selection of subjects given the large variability in corticomuscular coherence between subjects or that we only recorded EEG from a single electrode placed over Cz. It is therefore possible that we did not record from the most optimal position in all subjects.

Although it seems most likely that the observed increase in coherence in the beta and gamma bands is related to corticospinal activity, other possibilities should also be taken into account. We certainly cannot exclude that oscillations in other descending pathways, local spinal circuitries, and sensory feedback pathways at different levels throughout the nervous system contribute to the changes in coherence. This is not surprising given the dynamic and rhythmic nature of gait, which will elicit rhythmic oscillations in all sensori‐motor networks that contribute to the activation of the muscles. Brain imaging studies also confirm that sensory feedback mechanisms are responsible for a very significant part of the activation of cortical motor areas during locomotion (Christensen et al. 2000; Promjunyakul et al. 2015) and local inhibitory interneurons have been shown to modulate EEG‐EMG coherence in a sustained isometric contraction (Matsuya et al. 2017).

The increased oscillatory activity during visually guided walking may be related to a specific effect of visual information on corticospinal activity and thus reflect an increased contribution of corticospinal activity to the muscle activity during visually guided walking. This is consistent with findings in the cat showing directly that corticospinal neurons in the primary motor cortex increase their firing rate when the cat has to use visual information to place the paw carefully or when stepping over an obstacle (Amos et al. 1990; Drew 1993; Drew et al. 1996; Marple‐Horvat and Armstrong 1999). This is also in line with the observation that motor evoked potentials elicited by TMS of the primary motor cortex are increased during visually guided walking (Schubert et al. 1999). Koenraadt et al. (2014) failed to find any differences in blood flow to the primary motor cortex during visually guided walking as compared to normal treadmill walking using near‐infrared spectroscopy (Koenraadt et al. 2014). This is not surprising since the corticospinal neurons involved in generating corticomuscular (and intra‐ and intermuscular) coherence and motor evoked potentials to a given muscle constitute only a small proportion of the total number of neurons in the primary motor cortex. Increased beta coherence with increased attention has been observed for EEG‐EMG coherence in a isometric holding task (Kristeva‐Feige et al. 2002). It is not clear how changes in attention related to the task of hitting the visual targets modulates the central common drive in this study and it cannot be ruled out that the change in attention play a role in the modulation of central common drive.

Our observations are thereby directly relevant for improving gait performance during attention demanding tasks in patients with stroke and in elderly subjects (Mazaheri et al. 2014). Adequate processing of visual information during gait is essential in order to navigate in a challenging terrain and circumvent obstacles in order to avoid falling (Patla and Greig 2006; Drew and Marigold 2015). Gait training in neurological patients and elderly subjects therefore should not only involve simple treadmill training but also include specific training of the cognitive processing of visual information from the environment. Training which focuses specifically on visual attention and efficient cognitive processing of information from the environment during gait should be a central part of any rehabilitation program following brain lesion.

## Conclusion

We have observed increased central common drive to motor units in the same muscle and in synergistic muscles during visually guided walking, which is in all likelihood related to increased corticomuscular coherence. This indicates an increased corticospinal involvement in visually guided walking compared to normal walking.

## Conflict of interests

None declared.

## References

[phy213598-bib-0001] Amjad, A. M. , D. M. Halliday , J. R. Rosenberg , and B. A. Conway . 1997 An extended difference of coherence test for comparing and combining several independent coherence estimates: theory and application to the study of motor units and physiological tremor. J. Neurosci. Methods 73:69–79. 9130680 10.1016/s0165-0270(96)02214-5

[phy213598-bib-0002] Amos, A. , D. M. Armstrong , and D. E. Marple‐Horvat . 1990 Changes in the discharge patterns of motor cortical neurones associated with volitional changes in stepping in the cat. Neurosci. Lett. 109:107–112. 2314625 10.1016/0304-3940(90)90546-l

[phy213598-bib-0003] Barthelemy, D. , M. Willerslev‐Olsen , H. Lundell , B. A. Conway , H. Knudsen , F. Biering‐Sørensen , et al. 2010 Impaired transmission in the corticospinal tract and gait disability in spinal cord injured persons. J. Neurophysiol. 104:1167–1176. 20554839 10.1152/jn.00382.2010

[phy213598-bib-0004] Choi, J. T. , P. Jensen , and J. B. Nielsen . 2016 Locomotor sequence learning in visually guided walking. J. Neurophysiol. 115:2014–2020. 26864768 10.1152/jn.00938.2015 PMC4869504

[phy213598-bib-0005] Christensen, L. O. , P. Johannsen , T. Sinkjaer , N. Petersen , H. S. Pyndt , and J. B. Nielsen . 2000 Cerebral activation during bicycle movements in man. Exp. Brain Res. 135:66–72. 11104128 10.1007/s002210000493

[phy213598-bib-0006] Drew, T. 1991 Visuomotor coordination in locomotion. Curr. Opin. Neurobiol. 1:652–657. 1822312 10.1016/s0959-4388(05)80044-3

[phy213598-bib-0007] Drew, T. 1993 Motor cortical activity during voluntary gait modifications in the cat. I. Cells related to the forelimbs. J. Neurophysiol. 70:179–199. 8360715 10.1152/jn.1993.70.1.179

[phy213598-bib-0008] Drew, T. , and D. S. Marigold . 2015 Taking the next step: cortical contributions to the control of locomotion. Curr. Opin. Neurobiol. 33:25–33. 25643847 10.1016/j.conb.2015.01.011

[phy213598-bib-0009] Drew, T. , W. Jiang , B. Kably , and S. Lavoie . 1996 Role of the motor cortex in the control of visually triggered gait modifications. Can. J. Physiol. Pharmacol. 74:426–442. 8828889

[phy213598-bib-0010] Drew, T. , J. E. Andujar , K. Lajoie , and S. Yakovenko . 2008 Cortical mechanisms involved in visuomotor coordination during precision walking. Brain Res. Rev. 57:199–211. 17935789 10.1016/j.brainresrev.2007.07.017

[phy213598-bib-0011] Farmer, S. F. , F. D. Bremner , D. M. Halliday , J. R. Rosenberg , and J. A. Stephens . 1993 The frequency content of common synaptic inputs to motoneurones studied during voluntary isometric contraction in man. J. Physiol. 470:127–155. 8308721 10.1113/jphysiol.1993.sp019851 PMC1143910

[phy213598-bib-0012] Farmer, S. F. , J. Gibbs , D. M. Halliday , L. M. Harrison , L. M. James , M. J. Mayston , et al. 2007 Changes in EMG coherence between long and short thumb abductor muscles during human development. J. Physiol. 579:389–402. 17185340 10.1113/jphysiol.2006.123174 PMC2075402

[phy213598-bib-0013] Gerin‐Lajoie, M. , C. L. Richards , and B. J. McFadyen . 2005 The negotiation of stationary and moving obstructions during walking: anticipatory locomotor adaptations and preservation of personal space. Mot. Control 9:242–269. 10.1123/mcj.9.3.242 16239715

[phy213598-bib-0014] Gerin‐Lajoie, M. , C. L. Richards , J. Fung , and B. J. McFadyen . 2008 Characteristics of personal space during obstacle circumvention in physical and virtual environments. Gait Posture. 27:239–247. 17512201 10.1016/j.gaitpost.2007.03.015

[phy213598-bib-0015] Halliday, D. M. , S. F., Farmer, . 2010 On the need for rectification of surface EMG. J. Neurophysiol. 103: 3547; author reply 48‐9. 20530508 10.1152/jn.00222.2010

[phy213598-bib-0016] Halliday, D. M. , and J. R. Rosenberg . 2000 On the application, estimation and interpretation of coherence and pooled coherence. J. Neurosci. Methods 100:173–174. 11040381 10.1016/s0165-0270(00)00267-3

[phy213598-bib-0017] Halliday, D. M. , J. R. Rosenberg , A. M. Amjad , P. Breeze , B. A. Conway , and S. F. Farmer . 1995 A framework for the analysis of mixed time series/point process data–theory and application to the study of physiological tremor, single motor unit discharges and electromyograms. Prog. Biophys. Mol. Biol. 64:237–278. 8987386 10.1016/s0079-6107(96)00009-0

[phy213598-bib-0018] Halliday, D. M. , B. A. Conway , L. O. Christensen , N. L. Hansen , N. P. Petersen , and J. B. Nielsen . 2003 Functional coupling of motor units is modulated during walking in human subjects. J. Neurophysiol. 89:960–968. 12574472 10.1152/jn.00844.2002

[phy213598-bib-0019] Hansen, N. L. , B. A. Conway , D. M. Halliday , S. Hansen , H. S. Pyndt , F. Biering‐Sørensen , et al. 2005 Reduction of common synaptic drive to ankle dorsiflexor motoneurons during walking in patients with spinal cord lesion. J. Neurophysiol. 94:934–942. 15800077 10.1152/jn.00082.2005

[phy213598-bib-0020] James, L. M. , D. M. Halliday , J. A. Stephens , and S. F. Farmer . 2008 On the development of human corticospinal oscillations: age‐related changes in EEG‐EMG coherence and cumulant. Eur. J. Neurosci. 27:3369–3379. 18598272 10.1111/j.1460-9568.2008.06277.x

[phy213598-bib-0021] Koenraadt, K. L. , E. G. Roelofsen , J. Duysens , and N. L. Keijsers . 2014 Cortical control of normal gait and precision stepping: an fNIRS study. NeuroImage 85(Pt 1):415–422. 23631980 10.1016/j.neuroimage.2013.04.070

[phy213598-bib-0022] Kristeva‐Feige, R. , C. Fritsch , J. Timmer , and C. H. Lucking . 2002 Effects of attention and precision of exerted force on beta range EEG‐EMG synchronization during a maintained motor contraction task. Clin. Neurophysiol. 113:124–131. 11801434 10.1016/s1388-2457(01)00722-2

[phy213598-bib-0023] Krouchev, N. , and T. Drew . 2013 Motor cortical regulation of sparse synergies provides a framework for the flexible control of precision walking. Front. Comput. Neurosci. 7:83. 23874287 10.3389/fncom.2013.00083 PMC3708143

[phy213598-bib-0024] Lamontagne, A. , J. Fung , B. J. McFadyen , and J. Faubert . 2007 Modulation of walking speed by changing optic flow in persons with stroke. J. Neuroeng. Rehabil. 4:22. 17594501 10.1186/1743-0003-4-22 PMC1913055

[phy213598-bib-0025] Maeda, R. S. , S. M. O'Connor , J. M. Donelan , and D. S. Marigold . 2017 Foot placement relies on state estimation during visually guided walking. J. Neurophysiol. 117:480–491. 27760813 10.1152/jn.00015.2016 PMC5288482

[phy213598-bib-0026] Marigold, D. S. 2008 Role of peripheral visual cues in online visual guidance of locomotion. Exerc. Sport Sci. Rev. 36:145–151. 18580295 10.1097/JES.0b013e31817bff72

[phy213598-bib-0027] Marple‐Horvat, D. E. , and D. M. Armstrong . 1999 Central regulation of motor cortex neuronal responses to forelimb nerve inputs during precision walking in the cat. J. Physiol. 519(Pt 1):279–299. 10432358 10.1111/j.1469-7793.1999.0279o.x PMC2269495

[phy213598-bib-0028] Matsuya, R. , J. Ushiyama , and J. Ushiba . 2017 Inhibitory interneuron circuits at cortical and spinal levels are associated with individual differences in corticomuscular coherence during isometric voluntary contraction. Sci. Rep. 7:44417. 28290507 10.1038/srep44417 PMC5349562

[phy213598-bib-0029] Matthis, J. S. , and B. R. Fajen . 2014 Visual control of foot placement when walking over complex terrain. J. Exp. Psychol. Hum. Percept Perform. 40:106–115. 23750964 10.1037/a0033101

[phy213598-bib-0030] Mazaheri, M. , M. Roerdink , R. J. Bood , J. Duysens , P. J. Beek , and C. L. Peper . 2014 Attentional costs of visually guided walking: effects of age, executive function and stepping‐task demands. Gait Posture. 40:182–186. 24767613 10.1016/j.gaitpost.2014.03.183

[phy213598-bib-0031] McFadyen, B. J. , L. Bouyer , L. R. Bent , and J. T. Inglis . 2007 Visual‐vestibular influences on locomotor adjustments for stepping over an obstacle. Exp. Brain Res. 179:235–243. 17136529 10.1007/s00221-006-0784-0

[phy213598-bib-0032] Mohagheghi, A. A. , R. Moraes , and A. E. Patla . 2004 The effects of distant and on‐line visual information on the control of approach phase and step over an obstacle during locomotion. Exp. Brain Res. 155:459–468. 14770275 10.1007/s00221-003-1751-7

[phy213598-bib-0033] Nielsen, J. B. , B. A. Conway , D. M. Halliday , M. C. Perreault , and H. Hultborn . 2005 Organization of common synaptic drive to motoneurones during fictive locomotion in the spinal cat. J. Physiol. 569:291–304. 16166163 10.1113/jphysiol.2005.091744 PMC1464221

[phy213598-bib-0034] Nielsen, J. B. , J. S. Brittain , D. M. Halliday , V. Marchand‐Pauvert , D. Mazevet , and B. A. Conway . 2008 Reduction of common motoneuronal drive on the affected side during walking in hemiplegic stroke patients. Clin. Neurophysiol. 119:2813–2818. 18848803 10.1016/j.clinph.2008.07.283

[phy213598-bib-0035] Norton, J. A. , and M. A. Gorassini . 2006 Changes in cortically related intermuscular coherence accompanying improvements in locomotor skills in incomplete spinal cord injury. J. Neurophysiol. 95:2580–2589. 16407422 10.1152/jn.01289.2005

[phy213598-bib-0036] Patla, A. E. , and M. Greig . 2006 Any way you look at it, successful obstacle negotiation needs visually guided on‐line foot placement regulation during the approach phase. Neurosci. Lett. 397:110–114. 16413969 10.1016/j.neulet.2005.12.016

[phy213598-bib-0037] Patla, A. E. , and J. N. Vickers . 1997 Where and when do we look as we approach and step over an obstacle in the travel path? NeuroReport 8:3661–3665. 9427347 10.1097/00001756-199712010-00002

[phy213598-bib-0038] Patla, A. E. , and J. N. Vickers . 2003 How far ahead do we look when required to step on specific locations in the travel path during locomotion? Exp. Brain Res. 148:133–138. 12478404 10.1007/s00221-002-1246-y

[phy213598-bib-0039] Petersen, T. H. , M. Kliim‐Due , S. F. Farmer , and J. B. Nielsen . 2010 Childhood development of common drive to a human leg muscle during ankle dorsiflexion and gait. J. Physiol. 588:4387–4400. 20837641 10.1113/jphysiol.2010.195735 PMC3008846

[phy213598-bib-0040] Petersen, T. H. , M. Willerslev‐Olsen , B. A. Conway , and J. B. Nielsen . 2012 The motor cortex drives the muscles during walking in human subjects. J. Physiol. 590:2443–2452. 22393252 10.1113/jphysiol.2012.227397 PMC3424763

[phy213598-bib-0041] Petersen, T. H. , S. F. Farmer , M. Kliim‐Due , and J. B. Nielsen . 2013 Failure of normal development of central drive to ankle dorsiflexors relates to gait deficits in children with cerebral palsy. J. Neurophysiol. 109:625–639. 23136346 10.1152/jn.00218.2012

[phy213598-bib-0042] Prokop, T. , M. Schubert , and W. Berger . 1997 Visual influence on human locomotion. Modulation to changes in optic flow. Exp. Brain Res. 114:63–70. 9125452 10.1007/pl00005624

[phy213598-bib-0043] Promjunyakul, N. O. , B. D. Schmit , and S. M. Schindler‐Ivens . 2015 A novel fMRI paradigm suggests that pedaling‐related brain activation is altered after stroke. Front. Hum. Neurosci. 9:324. 26089789 10.3389/fnhum.2015.00324 PMC4454878

[phy213598-bib-0044] Schubert, M. , A. Curt , G. Colombo , W. Berger , and V. Dietz . 1999 Voluntary control of human gait: conditioning of magnetically evoked motor responses in a precision stepping task. Exp. Brain Res. 126:583–588. 10422722 10.1007/s002210050767

[phy213598-bib-0045] Storzer, L. , M. Butz , J. Hirschmann , O. Abbasi , M. Gratkowski , D. Saupe , et al. 2016 Bicycling and walking are associated with different cortical oscillatory dynamics. Front. Hum. Neurosci. 10:61. 26924977 10.3389/fnhum.2016.00061 PMC4759288

[phy213598-bib-0046] Winslow, A. T. , J. Brantley , F. Zhu , J. L. Contreras Vidal , and H. Huang . 2016 Corticomuscular coherence variation throughout the gait cycle during overground walking and ramp ascent: a preliminary investigation. Conf. Proc. IEEE Eng. Med. Biol. Soc. 2016:4634–4637. 28269308 10.1109/EMBC.2016.7591760

